# Supplementary material for: High-resolution ultrasonography for early diagnosis of neural impairment in seropositive leprosy household contacts
Source: PLoS One. 2023 May 23;18(5):e0285450. doi: 10.1371/journal.pone.0285450 (PMC10204990; doi:10.1371/journal.pone.0285450)
Supplement: S1 Table — ID: Patient identification; Upt: Ulnar nerve proximal to the cubital tunnel; Ut: Ulnar nerve at the cubital tunnel; Mpt: Median nerve proximal to the carpal tunnel; Mt: Median nerve at the carpal tunnel; Tpt: Tibial nerve proximal to the tarsal tunnel; Tt: Tibial nerve at the tarsal tunnel; NI: Measurement not included (evidence of carpal tunnel syndrome; previous history of fibula fracture). (DOCX) [file pone.0285450.s001.docx]

**Table S1. CSA measurements of each leprosy household contact included in the study.**

|  | **CSA measurements (mm²) for each nerve** | | | | | | | | | | | | | |
| --- | --- | --- | --- | --- | --- | --- | --- | --- | --- | --- | --- | --- | --- | --- |
| **ID** | **Right Upt** | **Right Ut** | **Right Mpt** | **Right Mt** | **Right common fibular** | **Right Tpt** | **Right Tt** | **Left Upt** | **Left Ut** | **Left Mpt** | **Left Mt** | **Left common fibular** | **Left Tpt** | **Left Tt** |
| 1 | 4 | 6 | 8 | 6 | 23 | 18 | 19 | 5 | 6 | 7 | 6 | 20 | 21 | 20 |
| 2 | 5 | 5 | 5 | 7 | 14 | 13 | 13 | 5 | 7 | 5 | 7 | 13 | 12 | 10 |
| 3 | 5 | 5 | 5 | 7 | 13 | 9 | 9 | 4 | 5 | 6 | 7 | 12 | 8 | 7 |
| 4 | 5 | 6 | 6 | 8 | 23 | 11 | 10 | 4 | 6 | 7 | 9 | NI | 10 | 10 |
| 5 | 6 | 5 | 5 | 9 | 16 | 10 | 10 | 5 | 6 | 5 | 7 | 17 | 12 | 9 |
| 6 | 4 | 6 | 5 | 5 | 14 | 8 | 8 | 4 | 5 | 7 | 8 | 13 | 8 | 9 |
| 7 | 6 | 5 | 6 | 7 | 14 | 10 | 10 | 6 | 5 | 6 | 8 | 14 | 10 | 10 |
| 8 | 4 | 6 | 6 | 7 | 14 | 8 | 6 | 5 | 7 | 6 | 7 | 13 | 7 | 7 |
| 9 | 5 | 5 | 7 | 8 | 20 | 12 | 12 | 5 | 6 | 7 | 8 | 20 | 9 | 9 |
| 10 | 5 | 5 | 5 | 5 | 16 | 11 | 11 | 6 | 6 | 6 | 7 | 13 | 10 | 10 |
| 11 | 5 | 5 | 8 | 9 | 11 | 9 | 8 | 5 | 6 | 7 | 9 | 12 | 8 | 9 |
| 12 | 6 | 6 | 6 | 11 | 11 | 10 | 10 | 6 | 6 | 7 | 11 | 12 | 10 | 10 |
| 13 | 6 | 7 | 6 | 7 | 15 | 11 | 11 | 5 | 7 | 6 | 8 | 15 | 12 | 11 |
| 14 | 4 | 4 | 4 | 5 | 12 | 6 | 7 | 4 | 5 | 4 | 5 | 11 | 7 | 8 |
| 15 | 5 | 5 | 7 | 5 | 12 | 9 | 9 | 5 | 6 | 7 | 7 | 13 | 9 | 9 |
| 16 | 5 | 6 | 5 | 10 | 25 | 11 | 11 | 6 | 6 | 6 | 7 | 20 | 8 | 9 |
| 17 | 6 | 8 | 6 | 10 | 15 | 16 | 16 | 5 | 8 | 7 | 11 | 14 | 15 | 15 |
| 18 | 5 | 5 | 5 | 7 | 12 | 9 | 9 | 5 | 5 | 6 | 7 | 12 | 11 | 9 |
| 19 | 5 | 6 | 5 | 7 | 10 | 10 | 10 | 5 | 6 | 6 | 7 | 10 | 9 | 9 |
| 20 | 4 | 8 | 6 | 8 | 15 | 10 | 10 | 5 | 8 | 6 | 7 | 17 | 12 | 12 |
| 21 | 4 | 4 | 5 | 7 | 12 | 11 | 10 | 4 | 5 | 5 | 7 | 13 | 11 | 9 |
| 22 | 4 | 4 | 6 | 8 | 14 | 10 | 12 | 4 | 5 | 5 | 8 | 12 | 10 | 10 |
| 23 | 4 | 5 | 7 | NI | 11 | 14 | 14 | 5 | 6 | 7 | NI | 11 | 11 | 11 |
| 24 | 7 | 8 | 7 | 9 | 14 | 11 | 12 | 8 | 8 | 6 | 8 | 34 | 11 | 12 |
| 25 | 5 | 7 | 6 | 8 | 15 | 11 | 12 | 7 | 6 | 6 | 8 | 12 | 11 | 12 |
| 26 | 7 | 6 | 8 | NI | 14 | 8 | 8 | 5 | 7 | 9 | 7 | 14 | 11 | 11 |
| 27 | 13 | 12 | 7 | NI | 17 | 19 | 18 | 9 | 14 | 9 | NI | 17 | 17 | 16 |
| 28 | 4 | 5 | 5 | 6 | 10 | 8 | 8 | 4 | 5 | 6 | 5 | 10 | 8 | 8 |
| 29 | 6 | 6 | 6 | NI | 11 | 10 | 8 | 5 | 6 | 6 | NI | 12 | 9 | 8 |
| 30 | 7 | 8 | 6 | 9 | 12 | 8 | 8 | 6 | 5 | 6 | 9 | 15 | 10 | 9 |
| 31 | 7 | 7 | 8 | 7 | 14 | 12 | 11 | 7 | 6 | 7 | 9 | 14 | 12 | 12 |
| 32 | 5 | 7 | 6 | 8 | 15 | 8 | 10 | 4 | 6 | 5 | 8 | 22 | 10 | 9 |
| 33 | 4 | 6 | 5 | 7 | 18 | 7 | 8 | 5 | 7 | 5 | 7 | 16 | 8 | 8 |
| 34 | 5 | 6 | 4 | 6 | 18 | 14 | 12 | 4 | 6 | 4 | 6 | 18 | 8 | 10 |
| 35 | 3 | 4 | 4 | 6 | 17 | 9 | 9 | 3 | 4 | 5 | 7 | 17 | 10 | 10 |
| 36 | 5 | 5 | 4 | 5 | 10 | 7 | 8 | 4 | 5 | 4 | 6 | 10 | 7 | 8 |
| 37 | 3 | 4 | 4 | NI | 16 | 9 | 8 | 4 | 5 | 5 | 7 | 16 | 8 | 9 |
| 38 | 4 | 5 | 6 | 8 | 13 | 7 | 8 | 4 | 5 | 6 | 7 | 13 | 7 | 7 |
| 39 | 5 | 5 | 5 | 6 | 12 | 7 | 8 | 5 | 5 | 6 | 7 | 20 | 10 | 11 |
| 40 | 4 | 6 | 5 | 6 | 10 | 8 | 8 | 4 | 6 | 4 | 5 | 10 | 8 | 8 |
| 41 | 6 | 8 | 5 | 6 | 11 | 8 | 8 | 7 | 7 | 5 | 6 | 12 | 7 | 9 |
| 42 | 5 | 5 | 5 | 6 | 10 | 9 | 8 | 5 | 5 | 5 | 7 | 12 | 8 | 8 |
| 43 | 4 | 4 | 7 | 7 | 10 | 8 | 8 | 5 | 4 | 6 | 6 | 10 | 8 | 8 |
| 44 | 3 | 4 | 6 | 6 | 12 | 8 | 9 | 3 | 4 | 7 | 7 | 12 | 9 | 8 |
| 45 | 5 | 6 | 6 | 6 | 12 | 8 | 8 | 5 | 6 | 7 | 7 | 11 | 9 | 9 |
| 46 | 4 | 6 | 6 | 7 | 12 | 10 | 10 | 4 | 5 | 6 | 7 | 13 | 9 | 9 |
| 47 | 4 | 6 | 5 | 7 | 9 | 6 | 7 | 4 | 6 | 4 | 6 | 8 | 7 | 7 |
| 48 | 4 | 5 | 5 | 6 | 8 | 8 | 7 | 4 | 5 | 4 | 5 | 8 | 8 | 8 |
| 49 | 6 | 7 | 8 | 9 | 24 | 9 | 8 | 5 | 6 | 7 | 8 | 23 | 10 | 10 |
| 50 | 4 | 5 | 6 | 10 | 13 | 10 | 8 | 4 | 6 | 7 | 9 | 13 | 10 | 10 |
| 51 | 5 | 5 | 10 | NI | 11 | 8 | 8 | 4 | 4 | 9 | 11 | 13 | 8 | 7 |
| 52 | 5 | 5 | 6 | 8 | 16 | 10 | 10 | 7 | 7 | 7 | 8 | 16 | 11 | 11 |
| 53 | 6 | 7 | 6 | 8 | 12 | 10 | 9 | 5 | 5 | 6 | 7 | 12 | 9 | 9 |
| 54 | 4 | 6 | 5 | 7 | 15 | 10 | 11 | 4 | 6 | 6 | 9 | 16 | 10 | 10 |
| 55 | 5 | 6 | 7 | 9 | 12 | 12 | 10 | 5 | 6 | 8 | 8 | 10 | 12 | 12 |
| 56 | 5 | 6 | 8 | 9 | 12 | 8 | 10 | 5 | 7 | 8 | 9 | 12 | 9 | 9 |
| 57 | 6 | 8 | 7 | 7 | 14 | 9 | 10 | 5 | 8 | 8 | 8 | 12 | 8 | 8 |
| 58 | 5 | 6 | 4 | 5 | 10 | 5 | 6 | 4 | 6 | 6 | 6 | 10 | 6 | 5 |
| 59 | 6 | 6 | 6 | 8 | 14 | 8 | 7 | 6 | 6 | 6 | 7 | 14 | 7 | 6 |
| 60 | 5 | 6 | 5 | 6 | 8 | 7 | 7 | 4 | 4 | 4 | 6 | 9 | 7 | 7 |
| 61 | 3 | 4 | 5 | 6 | 10 | 9 | 9 | 3 | 3 | 5 | 6 | 9 | 9 | 9 |
| 62 | 2 | 4 | 4 | 6 | 9 | 8 | 9 | 3 | 5 | 4 | 6 | 9 | 7 | 7 |
| 63 | 4 | 5 | 4 | NI | 14 | 8 | 9 | 4 | 5 | 4 |  | 14 | 9 | 10 |
| 64 | 5 | 6 | 7 | 7 | 12 | 10 | 10 | 5 | 6 | 7 | 7 | 12 | 10 | 10 |
| 65 | 5 | 6 | 6 | 8 | 11 | 8 | 8 | 5 | 6 | 5 | 7 | 11 | 8 | 8 |
| 66 | 4 | 6 | 5 | 7 | 12 | 7 | 7 | 4 | 6 | 5 | 7 | 12 | 6 | 6 |
| 67 | 5 | 7 | 8 | 10 | 11 | 10 | 10 | 6 | 7 | 7 | 9 | 11 | 10 | 10 |
| 68 | 3 | 3 | 4 | 5 | 8 | 6 | 6 | 4 | 4 | 4 | 5 | 8 | 5 | 5 |
| 69 | 5 | 6 | 6 | 8 | 11 | 9 | 9 | 5 | 6 | 6 | 8 | 11 | 8 | 9 |
| 70 | 5 | 6 | 5 | 7 | 11 | 9 | 9 | 5 | 6 | 5 | 6 | 11 | 9 | 9 |
| 71 | 4 | 5 | 5 | 7 | 12 | 10 | 9 | 4 | 5 | 5 | 7 | 12 | 10 | 9 |
| 72 | 3 | 3 | 5 | 6 | 12 | 8 | 9 | 3 | 4 | 4 | 5 | 12 | 9 | 8 |
| 73 | 5 | 5 | 7 | 8 | 10 | 10 | 11 | 5 | 6 | 6 | 7 | 10 | 10 | 11 |
| 74 | 6 | 7 | 6 | 8 | 10 | 7 | 7 | 5 | 6 | 6 | 8 | 10 | 7 | 7 |
| 75 | 4 | 4 | 6 | 8 | 11 | 9 | 9 | 5 | 5 | 6 | 7 | 11 | 9 | 9 |
| 76 | 4 | 5 | 7 | 8 | 10 | 8 | 8 | 4 | 5 | 7 | 8 | 10 | 8 | 8 |
| 77 | 4 | 5 | 6 | 8 | 12 | 9 | 9 | 3 | 4 | 6 | 7 | 12 | 10 | 10 |
| 78 | 5 | 7 | 7 | 8 | 14 | 8 | 9 | 6 | 7 | 6 | 7 | 15 | 7 | 8 |
| 79 | 5 | 6 | 6 | 8 | 14 | 9 | 8 | 5 | 6 | 6 | 8 | 14 | 9 | 10 |

Legend: ID: patient identification; Upt: ulnar nerve proximal to the cubital tunnel; Ut: ulnar nerve at the cubital tunnel; Mpt: median nerve proximal to the carpal tunnel; Mt: median nerve at the carpal tunnel; Tpt: tibial nerve proximal to the tarsal tunnel; Tt: tibial nerve at the tarsal tunnel; NI: measurement not included (evidence of carpal tunnel syndrome; previous history of fibula fracture).
