# Supplementary material for: High-resolution ultrasonography for early diagnosis of neural impairment in seropositive leprosy household contacts
Source: PLoS One. 2023 May 23;18(5):e0285450. doi: 10.1371/journal.pone.0285450 (PMC10204990; doi:10.1371/journal.pone.0285450)
Supplement: S2 Table — ID: Patient identification; ELISA: Enzyme-linked immunosorbent assay; anti-PGL-I: Anti-phenolic glycolipid I; ED: Extradomiciliary; ID: Intradomiciliary; PB: Paucibacillary; MB: Multibacillary; qPCR: Real Time Quantitative Polymerase Chain Reaction; BCG = Bacillus Calmette-Guérin. US: Ultrasonography. (DOCX) [file pone.0285450.s002.docx]

**Table S2. Clinical data of each leprosy household contact included in the study.**

| **ID** | **Gender** | **Age** | **ELISA anti-PGL-I** | **ELISA anti-PGL-I index** | **Type of contact** | **Index case** | **Neural thickening on clinical palpation** | **Skin biopsy qPCR** | **Slit-skin qPCR** | **BCG scars** | **US exam** |
| --- | --- | --- | --- | --- | --- | --- | --- | --- | --- | --- | --- |
| 1 | Female | 58 | Positive | 4.29 | ED | PB | No | Negative | Negative | 0 | Abnormal |
| 2 | Female | 57 | Positive | 2.27 | ED | PB | No | Negative | Negative | 0 | Abnormal |
| 3 | Male | 26 | Positive | 3.19 | ID | PB | No | Negative | Negative | 2 | Normal |
| 4 | Female | 68 | Positive | 2.01 | ED | MB | No | Negative | Negative | 0 | Abnormal |
| 5 | Female | 40 | Positive | 3.06 | ID | MB | No | Negative | Negative | 1 | Normal |
| 6 | Female | 60 | Positive | 1.53 | ID | MB | No | Negative | Negative | 2 | Normal |
| 7 | Female | 48 | Positive | 1.37 | ED | MB | No | Negative | Negative | 1 | Normal |
| 8 | Female | 62 | Positive | 1.7 | ID | MB | No | Negative | Positive | 0 | Normal |
| 9 | Male | 54 | Positive | 2.94 | ID | MB | No | Negative | Negative | 1 | Abnormal |
| 10 | Female | 31 | Positive | 1.04 | ID | MB | No | Positive | Positive | 1 | Normal |
| 11 | Female | 24 | Positive | 2.04 | ID | MB | No | Negative | Negative | 2 | Normal |
| 12 | Male | 37 | Positive | 1.67 | ED | MB | No | Negative | Negative | 1 | Normal |
| 13 | Female | 34 | Positive | 1.23 | ED | MB | No | Negative | Negative | 1 | Normal |
| 14 | Female | 16 | Positive | 2.86 | ED | MB | No | Negative | Negative | 1 | Normal |
| 15 | Female | 24 | Positive | 1.4 | ED | MB | No | Negative | Negative | 2 | Normal |
| 16 | Female | 42 | Positive | 1.42 | ID | MB | No | Negative | Negative | 1 | Abnormal |
| 17 | Female | 63 | Positive | 2.04 | ID | MB | No | Negative | Negative | 0 | Abnormal |
| 18 | Male | 48 | Positive | 3.63 | ID | MB | No | Negative | Negative | 0 | Normal |
| 19 | Male | 43 | Positive | 1.04 | ID | MB | No | Negative | Negative | 1 | Normal |
| 20 | Female | 41 | Positive | 2.07 | ED | MB | No | Negative | Negative | 1 | Normal |
| 21 | Female | 67 | Positive | 1.37 | ED | MB | No | Negative | Negative | 0 | Normal |
| 22 | Female | 16 | Positive | 2.28 | ID | MB | No | Negative | Negative | 1 | Normal |
| 23 | Female | 42 | Positive | 3.09 | ID | MB | No | Negative | Negative | 1 | Abnormal |
| 24 | Female | 58 | Positive | 2.67 | ED | MB | No | Negative | Negative | 1 | Abnormal |
| 25 | Male | 51 | Positive | 3 | ED | MB | No | Negative | Negative | 1 | Normal |
| 26 | Female | 61 | Positive | 1.15 | ID | MB | No | Negative | Negative | 1 | Normal |
| 27 | Female | 54 | Positive | 1.7 | ED | MB | Yes | Positive | Positive | 1 | Abnormal |
| 28 | Female | 21 | Positive | 1.58 | ID | MB | No | Negative | Negative | 1 | Normal |
| 29 | Female | 48 | Positive | 2.32 | ID | MB | No | Negative | Negative | 1 | Normal |
| 30 | Female | 63 | Positive | 2.14 | ID | MB | No | Negative | Negative | 0 | Normal |
| 31 | Female | 47 | Positive | 1.89 | ED | MB | No | Positive | Negative | 1 | Normal |
| 32 | Female | 34 | Positive | 1.71 | ED | MB | No | Negative | Negative | 2 | Abnormal |
| 33 | Female | 37 | Positive | 1.55 | ED | MB | No | Negative | Negative | 2 | Normal |
| 34 | Female | 63 | Positive | 3.5 | ID | MB | No | Negative | Positive | 0 | Abnormal |
| 35 | Female | 24 | Positive | 2.21 | ED | MB | No | Negative | Positive | 1 | Normal |
| 36 | Female | 24 | Positive | 1.47 | ED | MB | No | Negative | Positive | 2 | Normal |
| 37 | Female | 45 | Positive | 1.79 | ID | MB | No | Negative | Negative | 1 | Normal |
| 38 | Female | 32 | Positive | 2.66 | ED | MB | No | Negative | Negative | 2 | Normal |
| 39 | Male | 81 | Positive | 1.59 | ED | MB | No | Negative | Negative | 0 | Abnormal |
| 40 | Female | 54 | Positive | 1.04 | ID | MB | No | Negative | Negative | 1 | Normal |
| 41 | Male | 22 | Positive | 1.32 | ID | MB | No | Negative | Negative | 2 | Normal |
| 42 | Male | 29 | Positive | 2.37 | ED | MB | No | Negative | Negative | 2 | Normal |
| 43 | Female | 28 | Positive | 1.47 | ID | MB | No | Negative | Negative | 2 | Normal |
| 44 | Female | 23 | Positive | 1.73 | ID | MB | No | Negative | Negative | 2 | Normal |
| 45 | Male | 59 | Positive | 1.9 | ID | MB | No | Negative | Negative | 1 | Normal |
| 46 | Female | 28 | Positive | 1.44 | ID | MB | No | Negative | Negative | 2 | Normal |
| 47 | Female | 32 | Positive | 1.87 | ED | MB | No | Negative | Negative | 2 | Normal |
| 48 | Female | 17 | Positive | 1.98 | ID | MB | No | Negative | Negative | 1 | Normal |
| 49 | Female | 50 | Positive | 1.61 | ED | MB | No | Negative | Negative | 1 | Abnormal |
| 50 | Female | 57 | Negative | 0.04 | ED | MB | No | Negative | Negative | 1 | Normal |
| 51 | Female | 50 | Negative | 0.35 | ED | MB | No | Negative | Negative | 1 | Abnormal |
| 52 | Female | 39 | Negative | 0.2 | ED | MB | No | Negative | Negative | 2 | Normal |
| 53 | Female | 59 | Negative | 0.33 | ED | MB | No | Negative | Negative | 2 | Normal |
| 54 | Female | 26 | Negative | 0.29 | ED | MB | No | Negative | Negative | 2 | Normal |
| 55 | Male | 35 | Negative | 0.59 | ED | PB | No | Negative | Negative | 2 | Normal |
| 56 | Male | 70 | Negative | 0.27 | ED | MB | No | Negative | Positive | 0 | Normal |
| 57 | Female | 23 | Negative | 0.42 | ID | MB | No | Negative | Negative | 2 | Normal |
| 58 | Female | 25 | Negative | 0.12 | ED | MB | No | Negative | Negative | 2 | Normal |
| 59 | Female | 22 | Negative | 0.59 | ED | MB | No | Negative | Negative | 2 | Normal |
| 60 | Female | 26 | Negative | 0.64 | ED | MB | No | Negative | Negative | 2 | Normal |
| 61 | Male | 26 | Negative | 0.09 | ID | MB | No | Negative | Negative | 2 | Normal |
| 62 | Female | 40 | Negative | 0.71 | ID | PB | No | Negative | Negative | 1 | Normal |
| 63 | Female | 46 | Negative | 0.28 | ID | MB | No | Negative | Negative | 1 | Normal |
| 64 | Female | 52 | Negative | 0.47 | ID | MB | No | Negative | Negative | 2 | Normal |
| 65 | Female | 41 | Negative | 0.16 | ED | MB | No | Negative | Negative | 2 | Normal |
| 66 | Female | 55 | Negative | 0.38 | ID | MB | No | Negative | Negative | 1 | Normal |
| 67 | Male | 44 | Negative | 0.14 | ED | MB | No | Negative | Negative | 1 | Normal |
| 68 | Female | 16 | Negative | 0.27 | ID | MB | No | Negative | Negative | 1 | Normal |
| 69 | Female | 52 | Negative | 0.69 | ID | MB | No | Negative | Negative | 2 | Normal |
| 70 | Male | 54 | Negative | 0.71 | ED | MB | No | Positive | Positive | 2 | Normal |
| 71 | Female | 27 | Negative | 0.48 | ED | MB | No | Negative | Negative | 2 | Normal |
| 72 | Male | 55 | Negative | 0.13 | ID | MB | No | Negative | Negative | 1 | Normal |
| 73 | Male | 42 | Negative | 0.36 | ED | MB | No | Negative | Negative | 1 | Normal |
| 74 | Male | 31 | Negative | 0.69 | ID | MB | No | Negative | Negative | 2 | Normal |
| 75 | Male | 69 | Negative | 0.46 | ID | MB | No | Negative | Negative | 0 | Normal |
| 76 | Female | 70 | Negative | 0.25 | ED | MB | No | Negative | Negative | 0 | Normal |
| 77 | Female | 54 | Negative | 0.11 | ID | MB | No | Negative | Negative | 1 | Normal |
| 78 | Male | 30 | Negative | 0.26 | ED | MB | No | Negative | Negative | 2 | Normal |
| 79 | Male | 33 | Negative | 0.68 | ED | MB | No | Negative | Negative | 0 | Normal |

Legend: ID: patient identification; ELISA: enzyme-linked immunosorbent assay; anti-PGL-I: anti-phenolic glycolipid I; ED: extradomiciliary; ID: intradomiciliary; PB: paucibacillary; MB: multibacillary; qPCR: Real Time Quantitative Polymerase Chain Reaction; BCG = Bacillus Calmette-Guérin. US: ultrasonography.
