# Supplementary material for: High-resolution ultrasonography for early diagnosis of neural impairment in seropositive leprosy household contacts
Source: PLoS One. 2023 May 23;18(5):e0285450. doi: 10.1371/journal.pone.0285450 (PMC10204990; doi:10.1371/journal.pone.0285450)
Supplement: S3 Table — ID: Participant identification; Upt: Ulnar nerve proximal to the cubital tunnel; Ut: Ulnar nerve at the cubital tunnel; Mpt: Median nerve proximal to the carpal tunnel; Mt: Median nerve at the carpal tunnel; Tpt: Tibial nerve proximal to the tarsal tunnel; Tt: Tibial nerve at the tarsal tunnel. (DOCX) [file pone.0285450.s003.docx]

**Table S3. Epidemiological data and CSA measurements of each healthy volunteer included in the study.**

|  |  |  | **CSA measurements (mm²) for each nerve** | | | | | | | | | | | | | |
| --- | --- | --- | --- | --- | --- | --- | --- | --- | --- | --- | --- | --- | --- | --- | --- | --- |
| **ID** | **Gender** | **Age**  **(years)** | **Right Upt** | **Right Ut** | **Right Mpt** | **Right Mt** | **Right common fibular** | **Right Tpt** | **Right Tt** | **Left Upt** | **Left Ut** | **Left Mpt** | **Left Mt** | **Left common fibular** | **Left Tpt** | **Left Tt** |
| 1 | Female | 47 | 6 | 5 | 6 | 7 | 12 | 9 | 8 | 5 | 6 | 6 | 7 | 13 | 8 | 8 |
| 2 | Female | 40 | 5 | 5 | 7 | 7 | 11 | 8 | 7 | 4 | 7 | 8 | 8 | 10 | 8 | 8 |
| 3 | Male | 30 | 5 | 5 | 8 | 7 | 12 | 6 | 6 | 5 | 6 | 6 | 7 | 10 | 5 | 5 |
| 4 | Male | 28 | 6 | 7 | 7 | 8 | 13 | 7 | 8 | 6 | 7 | 7 | 8 | 10 | 9 | 10 |
| 5 | Male | 28 | 7 | 7 | 9 | 9 | 10 | 10 | 10 | 7 | 7 | 9 | 8 | 12 | 10 | 10 |
| 6 | Male | 30 | 5 | 6 | 7 | 7 | 13 | 10 | 10 | 6 | 5 | 6 | 7 | 11 | 9 | 8 |
| 7 | Male | 28 | 6 | 5 | 6 | 7 | 11 | 9 | 7 | 6 | 5 | 7 | 7 | 10 | 9 | 10 |
| 8 | Female | 38 | 5 | 6 | 7 | 6 | 12 | 9 | 7 | 4 | 4 | 6 | 8 | 11 | 8 | 7 |
| 9 | Female | 30 | 6 | 8 | 6 | 8 | 8 | 9 | 8 | 6 | 7 | 6 | 7 | 8 | 7 | 7 |
| 10 | Female | 34 | 5 | 8 | 7 | 9 | 15 | 10 | 10 | 6 | 6 | 6 | 8 | 15 | 10 | 10 |
| 11 | Male | 46 | 7 | 8 | 7 | 8 | 16 | 8 | 8 | 7 | 8 | 7 | 8 | 15 | 9 | 9 |
| 12 | Male | 27 | 4 | 4 | 5 | 6 | 10 | 9 | 10 | 4 | 4 | 6 | 7 | 10 | 10 | 10 |
| 13 | Female | 61 | 5 | 8 | 5 | 8 | 15 | 10 | 9 | 5 | 7 | 6 | 7 | 14 | 8 | 9 |
| 14 | Male | 44 | 5 | 7 | 7 | 9 | 15 | 10 | 10 | 5 | 7 | 6 | 9 | 14 | 8 | 8 |
| 15 | Female | 43 | 5 | 5 | 5 | 6 | 15 | 8 | 8 | 5 | 5 | 6 | 6 | 15 | 9 | 9 |
| 16 | Male | 26 | 4 | 6 | 6 | 8 | 12 | 8 | 8 | 6 | 6 | 5 | 7 | 13 | 9 | 10 |
| 17 | Male | 31 | 5 | 6 | 6 | 9 | 12 | 8 | 9 | 4 | 5 | 7 | 9 | 12 | 8 | 9 |
| 18 | Female | 48 | 3 | 5 | 6 | 7 | 15 | 9 | 9 | 3 | 4 | 5 | 6 | 15 | 10 | 10 |
| 19 | Female | 57 | 5 | 5 | 4 | 9 | 15 | 7 | 8 | 4 | 6 | 7 | 12 | 15 | 8 | 8 |
| 20 | Female | 57 | 6 | 7 | 5 | 10 | 15 | 9 | 8 | 6 | 8 | 5 | 9 | 15 | 10 | 10 |
| 21 | Female | 56 | 4 | 6 | 5 | 8 | 15 | 9 | 8 | 5 | 7 | 6 | 7 | 15 | 10 | 8 |
| 22 | Female | 56 | 4 | 7 | 6 | 8 | 13 | 7 | 7 | 5 | 8 | 6 | 7 | 13 | 7 | 7 |
| 23 | Female | 45 | 4 | 5 | 6 | 8 | 13 | 8 | 8 | 4 | 5 | 5 | 9 | 13 | 7 | 8 |
| 24 | Female | 50 | 3 | 4 | 6 | 8 | 16 | 8 | 9 | 3 | 5 | 5 | 8 | 16 | 8 | 8 |
| 25 | Female | 57 | 5 | 5 | 5 | 7 | 14 | 8 | 8 | 6 | 7 | 6 | 7 | 13 | 10 | 10 |
| 26 | Male | 29 | 6 | 6 | 7 | 8 | 14 | 11 | 9 | 6 | 6 | 7 | 9 | 14 | 10 | 10 |
| 27 | Male | 42 | 7 | 7 | 8 | 11 | 11 | 8 | 8 | 6 | 7 | 8 | 11 | 12 | 9 | 10 |
| 28 | Male | 26 | 5 | 6 | 7 | 7 | 11 | 9 | 10 | 5 | 7 | 8 | 9 | 12 | 8 | 8 |
| 29 | Female | 26 | 4 | 7 | 6 | 7 | 12 | 9 | 8 | 3 | 6 | 5 | 7 | 11 | 8 | 9 |
| 30 | Female | 50 | 5 | 5 | 7 | 7 | 14 | 10 | 9 | 7 | 8 | 9 | 10 | 13 | 10 | 10 |
| 31 | Female | 61 | 7 | 8 | 5 | 9 | 13 | 9 | 9 | 7 | 8 | 5 | 9 | 15 | 9 | 10 |
| 32 | Male | 21 | 5 | 6 | 6 | 8 | 10 | 8 | 8 | 5 | 5 | 7 | 7 | 9 | 7 | 7 |
| 33 | Female | 44 | 4 | 4 | 6 | 6 | 12 | 9 | 10 | 4 | 4 | 6 | 7 | 13 | 10 | 10 |
| 34 | Female | 46 | 5 | 7 | 6 | 11 | 15 | 10 | 11 | 6 | 8 | 5 | 9 | 15 | 10 | 11 |
| 35 | Female | 32 | 4 | 6 | 6 | 7 | 10 | 9 | 7 | 4 | 6 | 6 | 8 | 8 | 8 | 8 |
| 36 | Male | 27 | 5 | 6 | 5 | 8 | 13 | 10 | 8 | 6 | 7 | 5 | 8 | 14 | 10 | 10 |
| 37 | Female | 27 | 4 | 4 | 4 | 6 | 8 | 8 | 7 | 3 | 4 | 5 | 6 | 9 | 7 | 8 |
| 38 | Female | 39 | 4 | 7 | 5 | 8 | 9 | 6 | 6 | 4 | 6 | 5 | 7 | 8 | 8 | 7 |
| 39 | Female | 28 | 3 | 4 | 4 | 5 | 11 | 8 | 8 | 3 | 5 | 4 | 5 | 10 | 9 | 9 |
| 40 | Female | 58 | 4 | 4 | 5 | 7 | 11 | 8 | 7 | 3 | 4 | 5 | 6 | 12 | 9 | 8 |
| 41 | Female | 61 | 5 | 6 | 6 | 7 | 11 | 9 | 9 | 5 | 6 | 6 | 7 | 12 | 11 | 10 |
| 42 | Female | 35 | 4 | 4 | 6 | 7 | 11 | 8 | 9 | 4 | 5 | 5 | 7 | 10 | 9 | 9 |
| 43 | Male | 54 | 5 | 7 | 6 | 8 | 14 | 9 | 8 | 5 | 6 | 6 | 10 | 13 | 9 | 10 |
| 44 | Male | 42 | 4 | 4 | 6 | 7 | 10 | 8 | 8 | 5 | 5 | 7 | 7 | 9 | 8 | 7 |
| 45 | Male | 34 | 5 | 5 | 6 | 8 | 9 | 7 | 8 | 6 | 5 | 6 | 7 | 10 | 8 | 9 |
| 46 | Female | 26 | 4 | 5 | 4 | 5 | 9 | 5 | 6 | 4 | 4 | 4 | 6 | 9 | 6 | 5 |
| 47 | Female | 51 | 3 | 5 | 5 | 8 | 11 | 8 | 8 | 4 | 5 | 6 | 7 | 10 | 8 | 9 |
| 48 | Female | 65 | 5 | 6 | 7 | 9 | 12 | 7 | 8 | 5 | 7 | 7 | 11 | 13 | 8 | 8 |
| 49 | Male | 36 | 4 | 6 | 6 | 6 | 12 | 7 | 7 | 5 | 6 | 5 | 6 | 10 | 7 | 7 |
| 50 | Male | 43 | 5 | 6 | 6 | 7 | 13 | 9 | 10 | 4 | 5 | 5 | 7 | 12 | 9 | 10 |
| 51 | Female | 36 | 4 | 5 | 5 | 8 | 12 | 9 | 8 | 4 | 4 | 5 | 8 | 11 | 8 | 9 |
| 52 | Female | 42 | 4 | 5 | 5 | 6 | 10 | 7 | 7 | 3 | 4 | 4 | 5 | 11 | 8 | 9 |
| 53 | Female | 52 | 4 | 5 | 6 | 7 | 11 | 7 | 7 | 4 | 5 | 6 | 7 | 10 | 8 | 9 |

Legend: ID: participant identification; Upt: ulnar nerve proximal to the cubital tunnel; Ut: ulnar nerve at the cubital tunnel; Mpt: median nerve proximal to the carpal tunnel; Mt: median nerve at the carpal tunnel; Tpt: tibial nerve proximal to the tarsal tunnel; Tt: tibial nerve at the tarsal tunnel.
